# A Fragment Insertion of *AgDFR* Results in a White Flower Phenotype in *Arundina graminifolia* (Orchidaceae)

**DOI:** 10.3390/plants14111680

**Published:** 2025-05-31

**Authors:** Jie Li, Yonglu Wei, Jianpeng Jin, Jie Gao, Qi Xie, Fengxi Yang, Genfa Zhu

**Affiliations:** 1 Guangdong Key Laboratory of Ornamental Plant Germplasm Innovation and Utilization, Institute of Environmental Horticulture, Guangdong Academy of Agricultural Sciences, Guangzhou 510640, China; lijie@gdaas.cn (J.L.); weiyonglu@gdaas.cn (Y.W.); jinjianpeng@gdaas.cn (J.J.); xieqi@gdaas.cn (Q.X.);; 2 Guangdong Laboratory for Lingnan Modern Agriculture, Guangzhou 510640, China

**Keywords:** *Arundina graminifolia*, anthocyanins, white flower, dihydroflavonol 4-reductase, molecular markers

## Abstract

Bamboo orchid (*Arundina graminifolia*), a fast-growing evergreen terrestrial orchid with year-round flowering capacity, exhibits limited germplasm resources for white floral variants despite its ornamental significance. This study investigates the molecular basis of natural white flower formation through comparative analysis of purple- and white-flowered variants across bud, post-bud, and blooming stages. Histological examination revealed anthocyanin accumulation restricted to two to three upper epidermal cell layers in purple petals, while white petals showed complete pigment absence. Transcriptome profiling coupled with RT-qPCR validation identified eleven differentially expressed structural genes in anthocyanin biosynthesis. Notably, *AgDFR* expression remained undetectable across all white-flower developmental stages. Sequence analysis demonstrated identical 3030 bp promoter regions of *AgDFR* between two variants, while white-flower *AgDFR* coding sequences contained over 107 bp insertion after the 330th nucleotide, causing premature translation termination. Molecular marker validation confirmed the presence of a diagnostic 472 bp fragment in all colored variants (13 purple/pink lines) and its absence in white phenotypes. This study establishes that insertional mutagenesis in *AgDFR*’s coding region underlies natural white flower in *A. graminifolia*. The developed molecular marker enables reliable differentiation of white-flowered variants from pigmented counterparts, providing valuable tools for germplasm management and breeding programs.

## 1. Introduction

Flower color, a crucial ornamental trait in horticultural plants, is regulated by multiple intrinsic and extrinsic factors, including pigment composition and concentration in petals, epidermal cell morphological characteristics, and environmental variables [1]. In orchid flowers, the three main pigment groups are flavonoids (particularly anthocyanins as key chromogenic substances), carotenoids, and chlorophyll. Studies in *Phalaenopsis*, *Dendrobium*, *Cattleya*, and *Pleione limprichtii* have established that purple or black phenotype in petals/lips correlates with high cyanidin and delphinidin levels [1,2,3,4].

The anthocyanin biosynthesis pathway (ABP), a well-characterized secondary metabolic pathway in plants, initiates with phenylalanine conversion through sequential enzymatic reactions. Core structural genes encoding ABP enzymes—*CHS*, *CHI*, *F3H*, *F3*′*H*, *DFR*, *ANS*, and *UFGT*—have been functionally validated across plant species [5]. In orchids, differential expression of these genes directly regulates anthocyanin biosynthesis flux, determining floral pigmentation patterns. Transcriptional regulators, particularly MYB-bHLH-WD40 (MBW) complexes and light-responsive *HY5* factors, coordinately activate anthocyanin accumulation in pigmented tissues like petal epidermis and stem vasculature [3,6,7,8]. However, molecular mechanisms underlying natural white flower formation in orchids remain poorly understood.

Bamboo orchid (*Arundina graminifolia*), the sole species in genus *Arundina* (Orchidaceae), is predominantly distributed across tropical and subtropical regions spanning southern China, India, Thailand, Nepal, Malaysia, Singapore, Indonesia, and the Pacific Islands [9]. This medicinal orchid biosynthesizes pharmacologically active stilbenoids, with traditional applications in detoxification, anti-inflammatory therapies, and rheumatism treatment [10,11]. As a rapidly growing evergreen terrestrial orchid, it exhibits continuous flowering phenology peaking from September to January [9]. While wild populations predominantly show purple/pink flowers, natural white-flowered variants are exceptionally rare, with their genetic basis remaining uncharacterized.

This study conducted multi-stage comparative analyses of purple- and white-flowered *A. graminifolia* across bud, post-bud, and blooming stages. Integrating histological analysis with transcriptome profiling, we mapped spatiotemporal anthocyanin distribution and elucidated regulatory networks. Molecular cloning and sequence analysis of the differentially expressed *AgDFR* gene revealed its pivotal role in pigmentation divergence. Furthermore, we developed diagnostic molecular markers for white-flower identification based on *AgDFR* allelic variations. These findings establish a molecular framework for floral chromatic determination in *A. graminifolia* while providing genomic tools for precision breeding of ornamental traits.

## 2. Results

### 2.1. Flower Phenotypes and Spatial Localization of Pigments

**Figure 1:**
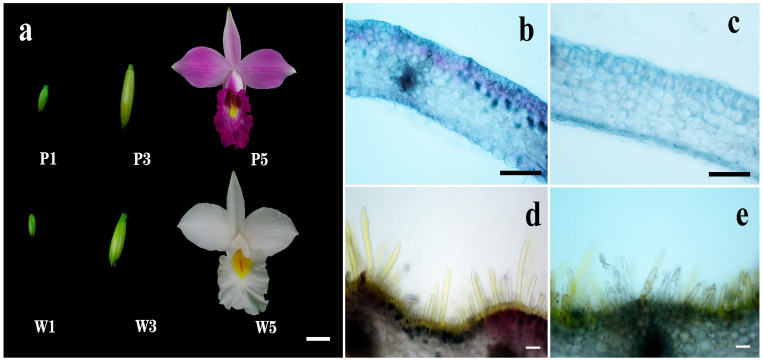
Three flower stages and pigment distribution of purple- and white-flowered *A. graminifolia*. (**a**): P1 and W1: bud stage, P3 and W3: post-bud stage, P5 and W5: full-blooming stage. Bar = 1 cm; (**b**,**c**): transverse section of petal in purple and white flower, respectively, Bar = 500 μm; (**d**,**e**): transverse section of lip in purple and white flower, respectively, Bar = 100 μm.

The *A. graminifolia* germplasms demonstrated distinct chromatic differentiation between floral morphotypes. Wild-type specimens exhibited uniform purple pigmentation distributed across petals, sepals, and labellum, featuring characteristic yellow maculae along the median labellar region (Figure 1a). In contrast, natural white-flowered variants maintained yellow labellar maculae while completely lacking anthocyanin pigmentation in other floral structures. Histological cross-sectional analysis revealed differential pigment distribution patterns: pigmented morphotypes showed localized anthocyanin deposition confined to two to three stratified upper epidermal cell layers, whereas white-flowered variants displayed complete absence of anthocyanin-containing chromoplasts. Both morphotypes exhibited yellow pigments accumulation within labellar papillate epidermal cells, with additional anthocyanin presence observed in the wild-type labellar epidermis (Figure 1b–e).

### 2.2. Overview of RNA Sequencing

According to transcriptome profiling data from five floral developmental stages [12], whole flowers at three key phases were collected: bud stage (P1 and W1), post-bud stage (P3 and W3), and full-bloom stage (P5 and W5) (Figure 1a). Comparative RNA-seq analysis of these tri-phasic developmental series between purple- and white-flowered morphotypes yielded 222.79 Gb of high-quality sequencing data (Q30 ≥ 91.52%; GC content: 48.79%). De novo assembly generated 302,722 unigenes with an average length of 856 bp (N50 = 1162 bp). BLAST-based annotation (NCBI BLAST 2.6.0+) revealed that all unigenes could be functionally assigned, with 129,336 (42.72%) showing significant matches in at least one reference database (Supplementary Tables S1–S3).

### 2.3. Identification of Differentially Expressed Genes (DEGs) in Anthocyanin Biosynthesis

**Figure 2:**
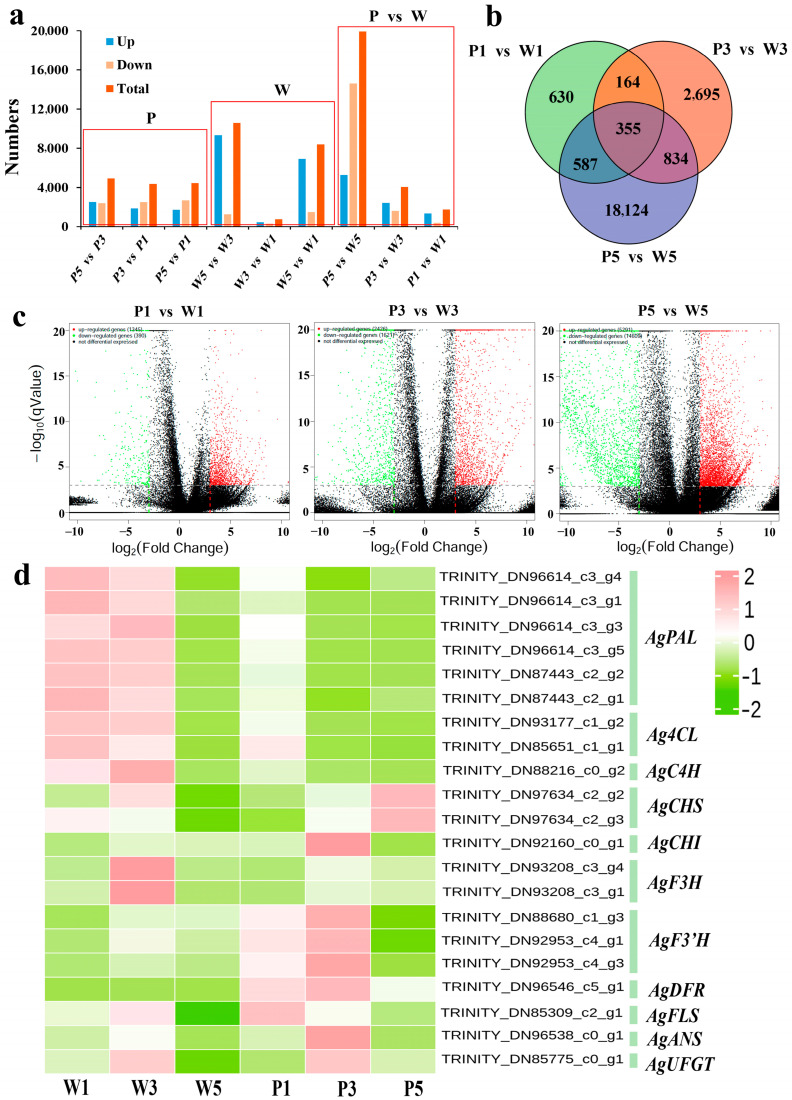
Analysis of differentially expressed genes (DEGs) derived from comparative transcriptomics among the different colors and flower stages. (**a**): Numbers of upregulated, downregulated, and total DEGs. W: Comparison group of different stages of white flowers. P: Comparison group of different stages of purple flowers. W_vs_P: Comparison group between white and purple flowers at the same stage. (**b**): Venn diagram displaying the DEGs among three comparison groups. (**c**): Volcano map diagram of DEGs in three comparison groups; red dots indicate upregulated DEGs, green dots indicate downregulated DEGs, and black dots indicate genes that did not show differential expression. (**d**): Heat map of DEGs involved in the anthocyanin biosynthesis. The text on the right side of the heatmap represents the transcript number and corresponding gene. P1, P3, and P5 represent bud, post-bud, and full-blooming stages of purple flowers; W1, W3, and W5 represent bud, post-bud, and full-blooming stages of white flowers.

Analysis of DEGs was performed between every two different samples, and nine sets of DEGs were identified from the W group, P group, and P vs. W group. The W and P groups represent the differential gene expression changes in white-flowered and purple-flowered *A. graminifolia* during the three flower stages. The number of DEGs in the P group was similar, while in the W group, W5 had more DEGs compared with W3 and W1, indicating that the formation of white flowers is complicated. In the P vs. W comparison, the number of DEGs increased gradually with flower development, and the number of DEGs in the W1-P1, W3-P3, and W5-P5 comparisons consisted of 1735 (390 downregulated and 1345 upregulated), 4047 (1621 downregulated and 2426 upregulated), and 19,900 (14,609 downregulated and 5291 upregulated) DEGs, respectively. Moreover, a total of 355 identical DEGs were found in the three comparison groups (Figure 2a–c).

Based on functional annotation and KEGG enrichment of DEGs, the DEGs in the P vs. W group were mapped to 361 various metabolic pathways, and 9, 11, and 1 DEGs were found to be involved in the phenylpropanoid biosynthesis (ko00940), flavonoid biosynthesis (ko00941), and anthocyanin biosynthesis (ko00942) pathways, respectively. The expression heat map of these twenty-one unigenes was listed in Figure 2d. The *AgPAL*, *Ag3CL*, and *AgC4H* genes were more highly expressed in white flowers than in purple flowers, especially in W1 and W3. The *AgCHI*, *AgF3′H*, *AgANS*, and *AgUFGT* genes exhibited increased expression at stage 3 (W3 and P3) in the two germplasms, and upregulated expression was detected in purple flowers compared with white flowers. Interestingly, the expression of the *AgDFR* gene significantly increased at the P3 stage with the development of purple flowers and then decreased at the P5 stage. However, in the white variant, this gene was almost not expressed at any of the three stages, which led to the inability to continue anthocyanin synthesis in white flowers. The *AgFLS* gene, which is a substrate competitor of *AgDFR*, was downregulated in purple flowers during flower development, but in white flowers, it was most highly expressed at the W3 stage.

### 2.4. Validation of the Expression of Anthocyanin-Related Genes

**Figure 3:**
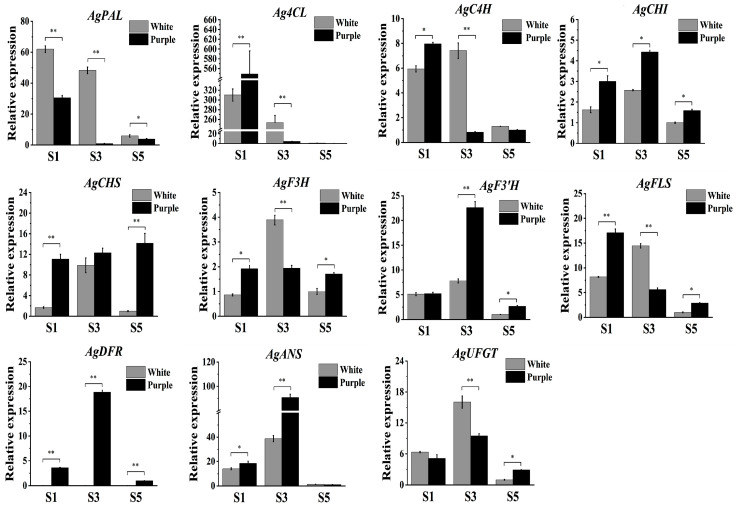
Validation of the expression of eleven genes. Significance analysis was performed using the *t*-test. * and ** indicate significant difference at *p* < 0.05 and *p* < 0.01 levels, respectively. S1 represents the bud stage for white and purple flowers. S3 represents the post-bud stage for white and purple flowers. S5 represents the full-blooming stage for white and purple flowers.

Eleven ABP-associated unigenes (Supplementary Table S4) underwent RT-qPCR validation across three developmental stages in both germplasms. Primer sequences are detailed in Supplementary Table S5. Expression profiles showed strong concordance with RNA-seq-derived transcripts per million (TPM) values, with *AgDFR* exhibiting no detectable expression throughout white flower development stages (Figure 3). This high correlation between sequencing and experimental data confirms methodological reliability.

### 2.5. Isolation of AgDFR Gene and Phylogenetic Analysis

**Figure 4:**
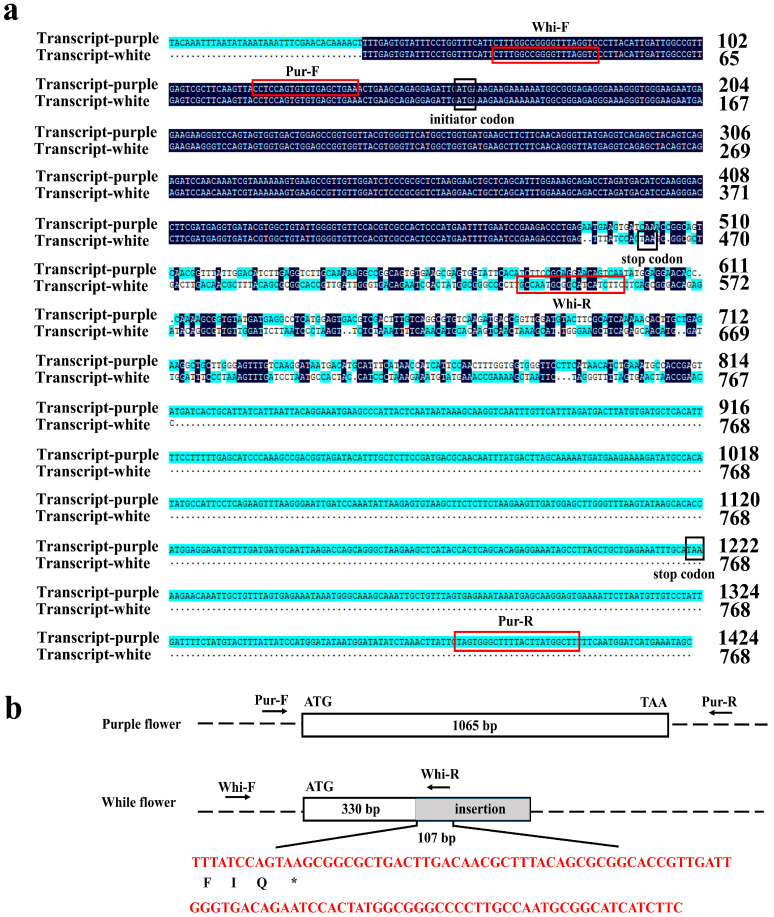
Sequence comparison of *AgDFR* gene in purple-flowered and white-flowered *A. graminifolia*. (**a**): Transcript sequence of *AgDFR* from white and purple flowers and primer position for gene cloning. Pur-F and Pur-R (in the red box) were primers for cloning in purple flowers; Whi-F and Whi-R (in the red box) were primers for cloning in white flowers. The sequence within the black box represents the gene’s start codon and stop codon. (**b**): Schematic illustration of the difference in *AgDFR* coding sequence, with a large fragment (more than 107 bp) inserted after the 330th nucleotide of the white flower’s *AgDFR*, resulting in early termination of the gene. * represents the stop codon.

To further investigate the role of the *AgDFR* gene in anthocyanin biosynthesis, its coding sequence was isolated using primers (Pur-F/Pur-R) designed according to the 1424 bp transcript. The *AgDFR* sequence from the cDNA of purple flowers contains a 1065 bp open reading frame (ORF) and encodes a deduced protein of 355 amino acids (Figure 4a,b). However, primers of Pur-F/Pur-R could not obtain PCR products from the cDNA of white flowers. Based on white flower’s sequencing data, we reassembled and gained a 768 bp transcript of the *AgDFR* gene. After redesigning the primers Whi-F/Whi-R, we obtained a 437 bp sequence from white flowers with an ORF (342 bp) encoding a deduced protein of 114 amino acids. According to the sequence alignment of the *AgDFR* gene coding sequence cloned from purple flowers and white flowers, it revealed that the *AgDFR* gene in white flowers had a sequence insertion greater than 107 bp after the 330th nucleotide, resulting in early termination of gene translation (Figure 4b).

**Figure 5:**
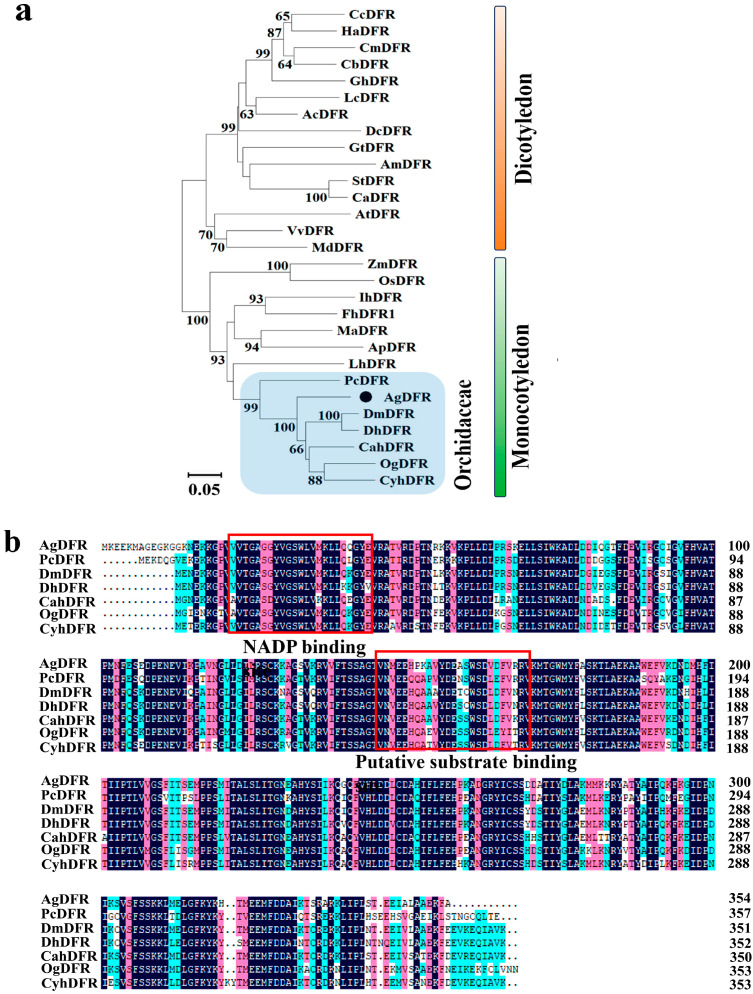
*AgDFR* gene phylogenetic tree and amino acid sequence alignment. (**a**): Phylogenetic relationships among DFR proteins from 29 plant species; numbers next to the nodes are bootstrap values from 1000 replications. The blue region represents the DFR proteins of Orchidaceae plants, while the small black circles denote the AgDFR proteins of *Arundina graminifolia*. (**b**): amino acid sequence alignment of *AgDFR* in seven Orchidaceae species. The putative NADP binding site and presumed substrate binding region are marked by red boxes. The GenBank accession numbers are as follows: *Oryza sativa* OsDFR (AB003495.1); *Zea mays* ZmDFR (Y16040.1); *Muscari armeniacum* MaDFR (AIC33028.1); *Lilium × hybrida* LhDFR (BAB40789.1); *Freesia hybrida* FhDFR (KU132393.1); *Agapanthus praecox* ApDFR (AB099529.1); *Iris × hollandica* IhDFR (BAF93856.1); *Actinidia chinensis* AcDFR (PSS36490.1); *Antirrhinum majus* AmDFR (X15536); *Arabidopsis thaliana* AtDFR (AB033294.1); *Callistephus chinensis* CcDFR (P51103.1); *Capsicum annuum* CaDFR (NP_001311706.1); *Chrysanthemum morifolium* CnDFR (ADC96612); *Daucus carota* DcDFR (XP_017254990.1); *Gentiana trifloral* GtDFR (BAA12736.1); *Gerbera hybrida* GhDFR (AKN56969.1); *Gynura bicolor* GbDFR (BAJ17657.1); *Helianthus annuus* HaDFR (XP_022001438.1); *Lonicera caerulea* LcDFR (ALU09329.1); *Solanum tuberosum* StDFR (AEN83503.1); *Vitis vinifera* VvDFR (X75964.1); *Malus domestica* MdDFR (AF117268.1); *Dendrobium moniliforme* DmDFR (HQ412559); *Dendrobium hybrid* DhDFR (FM209432.1); *Cattleya hybrid* CahDFR (KP171694.1); *Cymbidium hybrid* CyhDFR (KM186174.1); *Oncidium* Gower Ramsey OgDFR (AY953937.1); *Paphiopedilum concolor* PcDFR (JQ030890.1).

The phylogenetic analysis of 29 plant species showed that the DFRs of monocotyledons and dicotyledons were clearly divided into different branches. Seven DFR proteins, including AgDFR, were grouped into the same subclade in the family Orchidaceae, which indicated that these *DFR* genes of orchids have conserved functions in flavonoid biosynthesis. Amino acid sequence alignment of seven orchids revealed that *AgDFR* harbored a highly conserved NADP binding region and a substrate binding region at its N-terminus, and the similarities between the amino acid sequences of *AgDFR* and *PcDFR*, *DmDFR*, *DnDFR*, *OgDFR*, *CahDFR*, and *CyhDFR* were 77.92%, 85.59%, 84.46%, 83.48%, 80.47%, and 80.12%, respectively (Figure 5a,b).

### 2.6. Cloning of the AgDFR Gene Promoter

To verify the regulation of *AgDFR* expression by promoter differences, a 3030 bp sequence of the AgDFR promoter was cloned based on the designed primers. Only two SNPs (A/G) were found in −2782 bp and −2637 bp upstream of ATG between purple and white flowers, respectively, and neither SNP was located in a cis-acting regulatory element, indicating that the promoter sequence was not the cause of the difference in *AgDFR* expression (Supplementary Figure S1). Moreover, five MYB binding sites were found on the *AgDFR* promoter, indicating that MYB transcription factors may regulate *AgDFR* expression in the ABP of *A. graminifolia*. In addition, multiple cis-acting regulatory elements responding to abscisic acid, light, and methyl jasmonate were also found on the promoter of *AgDFR* (Supplementary Table S6).

### 2.7. Molecular Marker Identification and Proposed Model of White Flowers

**Figure 6:**
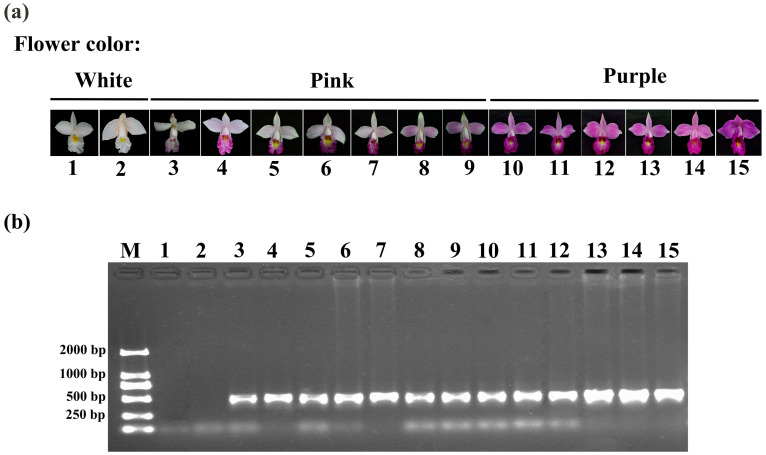
*AgDFR*-derived molecular markers for identification of white-flowered *Arundina graminifolia*. (**a**): Photographs of 15 *A. graminifolia* flowers showing different colors. (**b**): PCR products of *AgDFR* amplified using specific primers. M: DNA molecular weight markers. Lines 1 and 2 with white flowers lost the PCR fragment.

**Figure 7:**
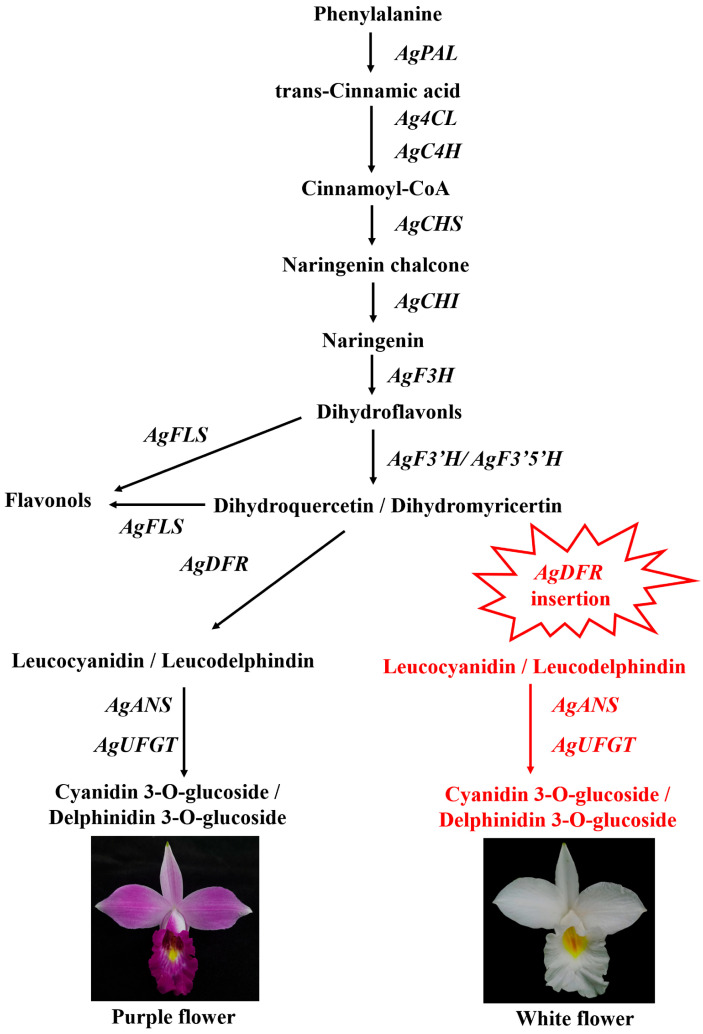
Molecular mechanism of different flower colors in *A. graminifolia*. A large fragment insertion in the coding region of the *AgDFR* gene leads to the loss of its function, disrupting the anthocyanin biosynthesis pathway and thereby resulting in the white flower phenotype. *AgDFR* and subsequent genes/compounds are highlighted in red font.

To develop molecular markers for subsequent identification of white-flowered A. graminifolia, this study designed specific primers targeting the AgDFR gene for genomic DNA amplification (Supplementary Table S5). The results showed that 13 pink-flowered and purple-flowered lines had a 472 bp fragment, but two white-flowered lines lost this fragment (Figure 6). Therefore, white-flowered *A. graminifolia* can be successfully distinguished from other colors using specific primers. These findings collectively demonstrate that molecular markers developed from the AgDFR gene can be utilized for subsequent identification of the white-flowered *A. graminifolia*. A schematic model illustrating the white-flower formation mechanism in *A. graminifolia* is presented in Figure 7.

## 3. Discussion

The current understanding of floral color evolution in angiosperms attributes color diversification primarily to natural selection pressures mediated by pollinator interactions, with white flower phenotypes predominantly resulting from anthocyanin biosynthesis deficiencies [13]. Although the etiology of floral albinism is multifactorial, the predominant mechanism involves functional impairment of structural genes in the anthocyanin pathway. In *Eustoma grandiflorum* and *Salvia miltiorrhiza*, frameshift deletions in the *ANS* gene have been shown to induce natural white-flower variants through premature termination of anthocyanin biosynthesis [14,15]. Similarly, a guanine-to-adenine nonsynonymous SNP (G/A) in the *DFR* coding region disrupts flavonoid 4-reductase activity, leading to the loss of anthocyanin accumulation in *Scutellaria baicalensis* [16]. In Orchidaceae, transcriptomic analyses of albescent variants in *Dendrobium nobile* [17], *Phalaenopsis amabilis* [2], and *Cymbidium kanran* [18] have revealed systemic downregulation of anthocyanin biosynthesis pathway structural genes, although the key regulatory determinants remain unidentified. Our study demonstrates that insertional mutagenesis in *AgDFR* disrupts canonical anthocyanin biosynthesis, establishing the molecular basis for natural white-flower phenotypes in *A. graminifolia*. Complementary evidence from *Iochroma loxense* shows that ectopic expression of R3-MYB transcriptional repressors can suppress pigmentation, generating white-flower phenotypes in transgenic tobacco [19]. These findings highlight the dual regulatory mechanisms underlying floral albinism, involving both structural gene mutations and transcriptional reprogramming.

Within the anthocyanin biosynthesis pathway, dihydroflavonol 4-reductase (DFR) serves as a pivotal downstream structural enzyme, catalyzing the stereospecific reduction of dihydroflavonols to chromatically inert leucoanthocyanidins. Critical to pigment diversification, *DFR* substrate specificity fundamentally determines anthocyanidin structural variation across plant species [20]. This enzyme competes with flavonol synthase (FLS) for dihydroflavonol substrates, with metabolic flux partitioning between flavonol and anthocyanin biosynthesis being governed by the relative expression levels of *FLS* and *DFR* [21]. Transgenic studies demonstrate that heterologous overexpression of *FLS* from *Rosa rugosa* or *Petunia hybrida* in tobacco enhances flavonol production while suppressing anthocyanin accumulation, culminating in floral albinism [22]. In *A. graminifolia*, we identified differential expression of upstream regulatory genes of *AgDFR* between the two floral color morphs. During the pre-anthesis post-bud stage, upstream genes *AgPAL*, *Ag4CL*, *AgC4H*, and *AgF3H* showed significant upregulation in white-flowered variants, potentially leading to enhanced accumulation of dihydroflavonol substrates. Given the loss-of-function of *AgDFR* coupled with sustained high expression of *AgFLS* during the post-bud stage in white flowers, we propose that dihydroflavonol flux is redirected toward flavonol biosynthesis. Conversely, purple-flowered morphs exhibited elevated expression of *AgDFR* and its downstream gene *AgANS* during the post-bud stage, facilitating increased anthocyanin accumulation. The distinct *AgFLS*-*AgDFR* co-expression patterns between purple- and white-flowered variants determine the metabolic fate of substrates toward either anthocyanin or flavonol biosynthesis. White-flowered plants demonstrated substantial flavonol compound accumulation, and subsequent metabolomic profiling will be performed to quantify flavonol metabolite divergence between the morphs.

*DFR* functions as a key gene in floral pigmentation. Mutations in this gene often lead to loss of pigmentation, thereby resulting in white flowers. In *Clarkia gracilis,* the *CgDFR* gene acts as a regulatory switch controlling purple spot pigmentation in petals [23]. In *Solanum* lineages, domestication-driven selection has shaped anthocyanin divergence through both alternative splicing of *DFR* transcripts, with a G→A single nucleotide polymorphism (SNP) within the second intron inducing premature termination codons and consequent floral albinism [24]. Similarly, C-terminal domain variations in *CmDFR* underlie chromatic divergence between red- and white-flowered *Chrysanthemum* cultivars during floral ontogeny [20]. Within Orchidaceae, comparative genomics reveals monogenic *DFR* status in *Cymbidium goeringii* and *Phalaenopsis equestris* [25,26], while our transcriptomic data confirm this monogenic architecture in *A. graminifolia*. Strikingly, while *AgDFR* promoter sequences (including core regulatory elements) are conserved between morphotypes, white-flowered variants exhibit over 107 bp insertion at nucleotide position 330 in the coding sequence, creating a frameshift mutation that truncates the functional protein. This loss-of-function mutation in *AgDFR* constitutes the molecular basis for natural white-flower phenotypes. Collectively, these findings establish *DFR* as a master regulator of floral pigmentation patterning across angiosperms.

## 4. Materials and Methods

### 4.1. Isolation of the AgDFR Promoter

Plants of *Arundina graminifolia* were grown in the germplasm resource nursery of the Guangdong Academy of Agricultural Sciences (Guangzhou, China). Mature plants with white flowers were collected from Dongguan Botanical Garden (China), while plants with purple and pink flowers and their hybrids were collected from Tibet and Shaoguan (China). Finally, a white-flower line and a purple-flower line were selected as the research materials for tissue section observation and transcriptome sequencing. According to previous transcriptome profiling of five flower stages [12], whole flowers at three developmental stages were sampled for transcriptome sequencing, gene expression, and cloning analysis, including bud (P1 and W1), post-bud (P3 and W3), and the full-blooming stage (P5 and W5). Fifteen lines of *A. graminifolia* (two with white flowers, seven with pink flowers, and six with purple flowers) were used for molecular marker analysis.

### 4.2. Tissue Section Observation

Whole petals and lips were separated from fresh flowers (W5 and P5 stage) and immersed immediately in a 0.25% polyethylene glycol solution (molecular weight: 8000, Sangon Biotech, Shanghai, China). Then, tissue sections were prepared according to the method described by Mudalige et al. [27], and the distribution of pigments was examined using a Nikon eclipse Ti-s microscope with Nikon camera attachment.

### 4.3. RNA Extraction, Library Preparation and Sequencing

Whole buds or flowers at three stages were sampled from white- and purple-flowered germplasms, frozen in liquid nitrogen, and preserved at −80 °C. RNA was extracted using a FastPure Universal Plant Total RNA Isolation Kit (Vazyme, Nanjing, China) with three biological replicates. A total of 18 RNA-seq libraries were constructed from these samples. RNA-Seq libraries were generated using the VAHTSTM mRNA-seq V2 Library Prep Kit for Illumina^®^ following the manufacturer’s recommendations. The library fragments were purified with an AMPure XP system (Beckman Coulter, Beverly, CA, USA). Library quality was assessed on an Agilent Bioanalyzer 2100 system. Paired-end sequencing of the library was performed on HiSeq X Ten sequencers (Illumina, San Diego, CA, USA).

### 4.4. De Novo Transcriptome Assembly Annotation

The clean reads were de novo assembled into transcripts using Trinity (version 2.0.6). Transcripts with a minimum length of 200 bp were clustered to minimize redundancy, and for each cluster, the longest sequence was preserved and designated a unigene. The unigenes were subjected to BLAST searches against the NCBI Nr (NCBI nonredundant protein database), SwissProt, TrEMBL, CDD (Conserved Domain Database), Pfam, and KOG (eukaryotic Orthologous Groups) databases (E-value < 1 × 10^−5^). TransDecoder (version 3.0.1) was used to predict the CDS of the unaligned unigenes. GO (Gene Ontology database) functional annotation information was obtained according to the transcript annotation results of the SwissProt and TrEMBL databases. KAAS (version 2.1) (KEGG Automatic Annotation Server) was used for KEGG (Kyoto Encyclopedia of Genes and Genomes) annotation.

### 4.5. Functional Analysis of Differentially Expressed Genes

Salmon (version 0.8.2) was used to calculate the read counts and expression values of the unigenes. The TPM (transcripts per million) eliminates the influence of gene length and sequencing discrepancies to enable direct comparisons of gene expression between samples. DESeq2 (version 1.12.4) was used to determine DEGs between two samples. Genes were considered significantly differentially expressed if the q value was <0.001 and |fold change| was >2. Gene Ontology (GO) and Kyoto Encyclopedia of Genes and Genomes (KEGG) analyses were performed to identify which DEGs were significantly enriched in GO terms or metabolic pathways, and a false discovery rate (q-value) < 0.05 was considered to indicate a significant difference.

### 4.6. Cloning of AgDFR

cDNA was synthesized by using the HiScript III 1st Strand cDNA Synthesis Kit (Vazyme, Nanjing, China). According to the *AgDFR* sequence, the Pu-F/R and Wh-F/R primer pairs were designed with Primer Premier 5.0 software (Supplementary Table S5) and used for cloning the *AgDFR* coding sequence from the cDNA of purple flowers and white flowers. PCR products were purified by the FastPure^®^ Gel DNA Extraction Mini Kit (Vazyme, Nanjing, China) and connected to the pCE2 TA/Blunt-Zero Vector (Vazyme, Nanjing, China) for transformation into DH5a (Weidibio, Shanghai, China). Finally, positive clones were selected for identification and Sanger sequencing.

### 4.7. Promoter Cloning

Approximately 3030 bp of the promoter sequence was selected and cloned for *AgDFR*, and the primers used are listed in Supplementary Table S5. The cis-acting elements and binding sites were predicted using the online tools PlantCARE “https://bioinformatics.psb.ugent.be/webtools/plantcare/html/ (accessed on 10 December 2023)” and PlantPan3.0 “http://plantpan.itps.ncku.edu.tw (accessed on 10 December 2023)”.

### 4.8. Multiple Sequence Alignment and Phylogenetic Analysis

The amino acid sequences of other DFR proteins in *A. thaliana* and other plant species were downloaded from the NCBI databases and aligned with *AgDFR* to construct a neighbor-joining phylogenetic tree in MEGA11, and the bootstrap consensus tree was inferred from 1000 replicates. Amino acid sequence alignment of DFRs was performed for seven orchids using DNAMAN (v.6.0) software 4.9.

### 4.9. Transcription Profiling by RT-qPCR

The expression of eleven key genes at the three stages of the two germplasms was measured using RT-qPCR. All primers used in this study are listed in Supplementary Table S5. RT-qPCR was conducted on a qTOWET 2.0 Real-Time PCR instrument (Jena, Germany) using SYBR qPCR Master Mix (Vazyme, Nanjing, China). Each reaction was performed using a 20 μL mixture containing 10 μL of 2× ChamQ SYBR^®^ qPCR Master Mix, 2 μL of cDNA, 6.4 μL of double-distilled water, and 0.8 μL of each primer (10 μM). The PCR conditions were 95 °C for 5 min, 40 cycles of amplification (95 °C for 15 s, 60 °C for 30 s, and 72 °C for 30 s), and 72 °C for 5 min. Three biological replicates were performed, and each reaction was repeated three times. The reference gene (actin gene) was used as an internal expression control, and the relative expression levels of the target genes were calculated by the 2^−ΔΔCt^ method [24].

### 4.10. Molecular Marker Identification

To develop molecular markers for subsequent identification of white-flowered *A. graminifolia*, we designed specific primers to amplify the 330 bp region of *AgDFR* using primer combinations (Supplementary Table S5). DNA was extracted from 15 lines of *A. graminifolia* using the FastPure Plant DNA Isolation Mini Kit-BOX2 (Vazyme, Nanjing, China). The PCR parameters were as follows: 95 °C for 3 min; 35 cycles of 95 °C for 15 s, 57 °C for 15 s, 72 °C for 45 s, and a final step of 72 °C for 5 min. The PCR products were separated by agarose gel electrophoresis.

## Data Availability

Data are contained within the article and Supplementary Materials, and all the raw RNA sequencing data have been submitted to the NCBI Sequence Read Archive (SRA) database under the accession number PRJNA1112030. The sequence of the *AgDFR* gene has been uploaded to GenBank (accession number PV566944).

## Supplementary Materials

The following supporting information can be downloaded at: https://www.mdpi.com/article/10.3390/plants14111680/s1, Figure S1: Differential fragment of *AgDFR* gene promoter; Table S1: Sequencing data statistics; Table S2: Statistics of transcript and unigene; Table S3: Gene annotation statistics; Table S4: Unigenes identified by RT-qPCR; Table S5: Primers for RT-qPCR and cloning; Table S6: Cis-acting element in *AgDFR* gene promoter.

## Abbreviations

The following abbreviations are used in this manuscript:

ABP	Anthocyanin biosynthesis pathway
DEGs	Differentially expressed genes
*CHS*	Chalcone synthase
*CHI*	Chalcone isomerase
*F3H*	Flavanone 3-hydroxylase
*F3′H*	Flavonoid 3′-hydroxylase
*F3′**5′**H*	Flavonoid 3′,5′-hydroxylase
*DFR*	Dihydroflavonol 4-reductase
*ANS*	Anthocyanidin synthase
*UFGT*	Anthocyanidin 3-O-glucosyltransferase
*PAL*	Phenylalanine ammonia-lyase
*4CL*	4-coumarate-CoA ligase
*C4H*	Cinnamic acid 4-hydroxylase
*FLS*	Flavonol synthase
*MYB*	v-myb avian myeloblastosis viral oncogene homolog
*bHLH*	Basic helix-loop-helix
*HY5*	Elongated hypocotyl 5
ORF	Open reading frame
