# Supplementary material for: A Fragment Insertion of AgDFR Results in a White Flower Phenotype in Arundina graminifolia (Orchidaceae)
Source: Plants (Basel). 2025 May 31;14(11):1680. doi: 10.3390/plants14111680 (PMC12157268; doi:10.3390/plants14111680)
Supplement: Supplementary file 1 [file plants-14-01680-s001.zip › Supplementary Figure.pdf]

```
White    TTAATCAAAAGATGTTACTCTTATCAACCCCACACATAACAACATACAAACATAGAAACCAACAAATAATTTTGGCTCATTTCTTTTTATATTTCAATAG   2100
Purple   TTAATCAAAAGATGTTACTCTTATCAACCCCACACATAACAACATACAAACATAGAAACCAACAAATAATTTTGGCTCATTTCTTTTTATATTTCAATAG   2100
Consensusttaatcaaaagatgttactcttatcaaccccacacataacaacatacaaacatagaaaccaacaaataattttggctcatttctttttatatttcaatag

White    ATGATATGATGCAATGAAAATTATGCAAAAGATAATTCTAAGTTAATACAAATGTTAATATAATGTTGTAGTAAGTAGTATCTTTCCAATGCTTAACTTA   2200
Purple   ATGATATGATGCAATGAAAATTATGCAAAAGATAATTCTAAGTTAATACAAATGTTAATATAATGTTGTAGTAAGTAGTATCTTTCCAATGCTTAACTTA   2200
Consensusatgatatgatgcaatgaaaattatgcaaaagataattctaagttaatacaaatgttaatataatgttgtagtaagtagtatctttccaatgcttaactta

White    AAACAAGATTTGCACAAACATGAAAATGACTTTATTAAACATGAAAGACCATGAATTCAAATTAAAAAAAACATTTTCTGATAAACCATGACATTAAAAT   2300
Purple   AAACAAGATTTGCACAAACATGAAAATGACTTTATTAAACATGAAAGACCATGAATTCAAATTAAAAAAAACATTTTCTGATAAACCATGACATTAAAAT   2300
Consensusaaacaagatttgcacaaacatgaaaatgactttattaaacatgaaagaccatgaattcaaattaaaaaaaacattttctgataaaccatgacattaaaat

White    CAAAAGTAAGGTGCAAGAAAAAAAGATTGAAATACAAATAGCAAAAACTATTTAGAAATAATTAGAGTTTGGAACAAATATCACCAAGGCAAGTTATAGT   2400
Purple   CAAAAGTAAGGTGCAAGAAAAAAAGATTGAAATACAAATAGCAAAAACTATTTAGAAATAATTAGAGTTTGGAACAAATATCACCAAGGCAAGTTATAGT   2400
Consensuscaaaagtaaggtgcaagaaaaaaagattgaaatacaaatagcaaaaactatttagaaataattagagtttggaacaaatatcaccaaggcaagttatagt

White    CTCCAAGATGAATGAATTTAACTAATGAATTCTTCATGGAGATGAAGCTTTGAATTCTTCTTACAAAAAGACTTGAAGAGATGGAGCAATAACTGTAACT   2500
Purple   CTCCAAGATGAATGAATTTAACTAATGAATTCTTCATGGAGATGAAGCTTTGAATTCTTCTTACAAAAAGACTTGAAGAGATGGAGCAATAACTGTAACT   2500
Consensusctccaagatgaatgaatttaactaatgaattcttcatggagatgaagctttgaattcttcttacaaaaagacttgaagagatggagcaataactgtaact

White    AATGGCTAAATAAAATTGGAGGTATTAGGGTTTTGCAAGAGAGGTAAGAATGTGACGTCGATGGTCATATGGCCCAAAGCAAAAAAAAAAAAAAAAAAGA   2600
Purple   AATGGCTAAATAAAATTGGAGGTATTAGGGTTTTGCAAGAGAGGTAAGAATGTGACGTCGATGGTCATATGGCCCAAAGCAAAAAAAAAAAAAAAAAAGA   2600
Consensusaatggctaaataaaattggaggtattagggttttgcaagagaggtaagaatgtgacgtcgatggtcatatggcccaaagcaaaaaaaaaaaaaaaaaaga

White    AGAAATAATCATTTAATTAATTTGAGAATTATTTTTAAATGCCTGCAATGCTATGATTCATAACGTGGCATCAAGGAATTGAAATTAAGCCAAAATCTAA   2700
Purple   AGAAATAATCATTTAATTAATTTGAGAATTATTTTTGAATGCCTGCAATGCTATGATTCATAACGTGGCATCAAGGAATTGAAATTAAGCCAAAATCTAA   2700
Consensusagaaataatcatttaattaatttgagaattattttt aatgcctgcaatgctatgattcataacgtggcatcaaggaattgaaattaagccaaaatctaa

White    AGTTGTTTTAGAAATAAAACCTCATAAAAGCCTTACTCAGTTATGGTTCCCCCTAAGGAAGGTTAGTGTATCTAAAATGCTATACTTTCAACTTTTCTAG   2800
Purple   AGTTGTTTTAGAAATAAAACCTCATAAAAGCCTTACTCAGTTATGGTTCCCCCTAAGGAAGGTTAGTGTATCTAAAATGCTGTACTTTCAACTTTTCTAG   2800
Consensusagttgttttagaaataaaacctcataaaagccttactcagttatggttccccctaaggaaggttagtgtatctaaaatgct tactttcaacttttctag

White    GAGATGTCACTCATTTGTTGCTTTATCTAAACACTTGCAAAAGTGTAAATGCAGCATTTGACTAATTCTCTAATGTTAGCTGCTCATATCATGAGGAAAC   2900
Purple   GAGATGTCACTCATTTGTTGCTTTATCTAAACACTTGCAAAAGTGTAAATGCAGCATTTGACTAATTCTCTAATGTTAGCTGCTCATATCATGAGGAAAC   2900
Consensusgagatgtcactcatttgttgctttatctaaacacttgcaaaagtgtaaatgcagcatttgactaattctctaatgttagctgctcatatcatgaggaaac

White    ATTTAAATCTCTCTCTATACCGCAACTATATGCGAATCTCTTGGAGCCTTATTGGGCCAAATTAATAAAGAAAAGAGAATGTACTCTAAGCCGTCGGTTG   3000
Purple   ATTTAAATCTCTCTCTATACCGCAACTATATGCGAATCTCTTGGAGCCTTATTGGGCCAAATTAATAAAGAAAAGAGAATGTACTCTAAGCCGTCGGTTG   3000
Consensusatttaaatctctctctataccgcaactatatgcgaatctcttggagccttattgggccaaattaataaagaaaagagaatgtactctaagccgtcggttg

White    CTTAAGGGCGAATTCGCGGCCGCTAAATCA                                                                         3030
Purple   CTTAAGGGCGAATTCGCGGCCGCTAAATCA                                                                         3030
Consensuscttaagggcgaattcgcggccgctaaatca
```

Figure S1. Differential fragment of *AgDFR* gene promoter
